# Vitamin D deficiency as adverse drug reaction? A cross-sectional study in Dutch geriatric outpatients

**DOI:** 10.1007/s00228-016-2016-2

**Published:** 2016-02-12

**Authors:** A. C. B. van Orten-Luiten, A. Janse, R. A. M. Dhonukshe-Rutten, R. F. Witkamp

**Affiliations:** Division of Human Nutrition, Pharmacology and Nutrition, Wageningen University, P.O. Box 8129, 6700 EV Wageningen, The Netherlands; Department of Geriatric Medicine, Gelderse Vallei Hospital, Willy Brandtlaan 10, 6716 RP Ede, The Netherlands; Division of Human Nutrition, Wageningen University, P.O. Box 8129, 6700 EV Wageningen, The Netherlands

**Keywords:** Adverse drug reaction, Drug-food interaction, Vitamin D, Polypharmacy, Elderly

## Abstract

**Purpose:**

Adverse drug reactions as well as vitamin D deficiency are issues of public health concern in older people. However, relatively little is known about the impact of drug use on vitamin D status. Our primary aim is to explore associations between drug use and vitamin D status in older people. Furthermore, prevalences of drug use and vitamin D deficiency are estimated.

**Methods:**

In a population of 873 community-dwelling Dutch geriatric outpatients, we explored the cross-sectional relationships of polypharmacy (≥5 medications concomitantly used), severe polypharmacy (≥10 medications), and use of twenty-one specific drug groups, with serum 25-hydroxyvitamin D (25(OH)D) by analysis of covariance.

**Results:**

Overall prevalence of polypharmacy was 65 %, of severe polypharmacy 22 %. Depending on the cut-off value, prevalence of vitamin D deficiency was 49 % (<50 nmol/l) or 77 % (<75 nmol/l). Of the patients using a vitamin D supplement, 17 % (<50 nmol/l) or 49 % (<75 nmol/l) were still deficient. In non-users of supplemental vitamin D, after adjustment for age and gender, negative associations were found for severe polypharmacy, metformin, sulphonamides and urea derivatives (SUDs), vitamin K antagonists, cardiac glycosides, loop diuretics, potassium-sparing diuretics, ACE inhibitors, and serotonin reuptake inhibitors; for non-selective monoamine reuptake inhibitors (NSMRIs) the association was positive. The most extreme impacts of drug use on adjusted mean 25(OH)D were −19 nmol/l for SUDs and +18 nmol/l for NSMRIs.

**Conclusion:**

Drug use should be considered a risk factor for vitamin D deficiency amongst geriatric outpatients.

**Electronic supplementary material:**

The online version of this article (doi:10.1007/s00228-016-2016-2) contains supplementary material, which is available to authorized users.

## Introduction

Adverse drug reactions (ADRs) substantially contribute to global disease burden. Median prevalence of ADR-related hospital admissions in European countries is estimated 3.5 %, prevalence of ADRs amongst inpatients 10.1 % [1]. These figures increase significantly with increase in age [2].

The high prevalence of vitamin D deficiency in older people is also an issue of global public health concern [3]. Apart from a negative impact on calcium metabolism and bone health, vitamin D deficiency might also lead to non-skeletal diseases and an increased inflammatory status: [4, 5]. An important determinant of deficiency of this micronutrient is a reduced synthesis of precursors in the skin by increase in age, less exposure to sunlight, or pigmented skin. Furthermore, insufficient dietary intake, impaired absorption, liver or kidney dysfunction, obesity, and inflammation may be of influence [6–10]. However, about the role of medication little is known. Observational and experimental research on this subject is scarce [11]. Also during drug development and in post-marketing studies, drug effects on nutritional status are not specifically addressed [12]. This is remarkable, as drug use as well as an impaired nutritional status are related to frailty [13–15] and predict disability, hospitalisations, and death [16].

Challenged by the lack of knowledge of this type of adverse drug reaction, we formulated the following study objectives: to investigate cross-sectional relationships of polypharmacy, number of drugs used, and use of 21 individual drug groups with serum 25-hydroxyvitamin D (25(OH)D); secondary aims were to determine prevalences of drug use and vitamin D deficiency. For statistical analyses we used data from a population of community-dwelling geriatric outpatients of a Dutch, non-academic, regional hospital.

## Methods

### Study population

Our original study population consisted of 892 community-living outpatients of the department of Geriatric Medicine of Gelderse Vallei Hospital (Ede, The Netherlands) at their first visit of the outpatient clinic, from August 2011 until January 2013. Indications for consultation were cognitive problems (60 %), falls (14 %) or other (24 %). Patient data were registered in the electronic patient file system Norma EPD. After exclusion of subjects either younger than 55 years or not having 25(OH)D measured, data of 783 patients were left for initial analysis. In a subgroup of 631 patients not using a vitamin D supplement, associations between drug use and serum vitamin D were explored in a simple regression model. Because of missing data, another 29 subjects were omitted in the complex model (*N* = 602). According to the Dutch Medical Research Involving Human Subjects Act (WMO) no ethical approval was needed, since this study involved retrospective analysis of anonymised patient data only.

### Data

History, physical examination and other clinical assessments of the geriatric patients were conducted by the medical staff, assisted by trained nurses. Non-fasting blood samples were collected at the Gelderse Vallei Hospital on the day of first visit. Assays were conducted by the hospital laboratory once a week. Vitamin D status was measured using the 25-OH-Vitamin D3/D2 Reagent Kit for HPLC analysis according to the manufacturer’s instructions (Chromsystems Instruments & Chemicals, Gräfelfing, Germany). For more details on 25(OH)D assessment we refer to the electronic supplementary material. Standardised anthropometric measurements were conducted by trained nurses. Weight was determined to the nearest 0.1 kg through a digital floor scale (Seca 770), height to the nearest 0.1 cm using a telescopic height rod (Seca 220). If patients had difficulties with standing, only weight was measured using a chair scale (Seca D94-09-033). Assessment of drug use was based on both prescribed and over-the-counter drugs the patients were asked to bring to the clinic, on (hetero)anamnesis, and on medication lists from pharmacies, if available. Non-compliance was structurally asked for. The Mini Nutritional Assessment (MNA) was performed in patients aged 65 and older. Collected patient data were registered in the hospital electronic patient file system Norma/NeoZis according to hospital standards.

Raw patient data were extracted from the patient files and further processed. The day of blood collection was recorded into the variable season: winter (October–March) or summer (April–September). Education attainment was classified as either primary school (≤6 years) or post-primary education and higher (>6 years), smoking as never, ever, and current. Categorisation of alcohol use was based on the Alcohol Consumption Index according to Garretsen: not/light, moderate, excessive/very excessive [17]. Medication and supplements were coded by the Anatomic Therapeutical Classification (ATC) index. Polypharmacy was defined as the concomitant use of five or more different ATC-coded substances; severe polypharmacy for ten or more. Medications of interest were drug groups at ATC level 4 used by minimally 10 % of the patients: proton pump inhibitors (PPIs), osmotically active laxatives, biguanides, sulfonamides and urea derivatives (SUDs), vitamin K antagonists, platelet aggregation inhibitors, thiazide diuretics, loop diuretics, selective beta-blocking agents, dihydropyridines, angiotensin-converting enzyme (ACE) inhibitors, angiotensin-2 antagonists, statins, anilides, non-selective monoamine reuptake inhibitors (NSMRIs), and selective serotonin reuptake inhibitors (SSRIs). For a more complete comparison with the results of a similar cross-sectional study, also cardiac glycosides, benzodiazepines, oral antidiabetics, potassium-sparing diuretics, and antidepressants were investigated [18]. After being processed, data were ready to be analysed.

### Statistical analysis

Statistical analyses were conducted with IBM SPSS statistics Version 20. Because of non-normal distributions, numerical data were reported as medians with interquartile range (IQR); categorical data as prevalences. Medians were compared by the nonparametric median test, differences between prevalences by chi-squared tests. Analysis of covariance (ANCOVA) was applied to investigate the associations between the dependent variable 25(OH)D, and the independent variables ‘number of ATC-coded substances’, polypharmacy, severe polypharmacy, and twenty-one individual drug groups in subjects not taking a specific vitamin D supplement. Use of medication was compared to non-use.

Two regression models were constructed for each drug group in non-users of supplemental vitamin D. Firstly, a simple regression model with age and gender as confounders, and secondly, a complex model that also adjusts for BMI, MMSE score, and use of multivitamins. Pragmatically, one complex model was created for all drug groups by using ‘number of ATC-coded substances used’ as independent variable. Based on literature, the following variables were considered as extra, potential confounders: BMI, season, plasma creatinine, plasma albumin, MMSE score, education, smoking, use of alcohol, use of a vitamin D supplement, and use of a multivitamin supplement [18, 19]. Variables were included in the model if they changed the unstandardised regression coefficient ≥10 % after being added (linear regression, stepwise method).

To meet the assumption of normal distribution of the dependent variable and its residuals, square-root transformation of 25(OH)D was applied. Normality was checked through histograms, normal Q-Q plots, and detrended Q-Q plots. Since associations between medication and square-root transformed 25(OH)D values and differences in means of such a variable are difficult to interpret, the adjusted means of transformed 25(OH)D were squared (back transformation) to calculate the easy to interpret mean untransformed 25(OH)D levels of users and non-users of a medication. The difference in adjusted means 25(OH)D between users and non-users of a specific drug group is equivalent to the regression coefficient beta (β). This is important to realise as a regression coefficient, difference, or confidence interval of a square-root transformed variable cannot be squared as back transformation [20]. The assumption homogeneity of variance was tested with the Levene’s test. In case of significance, we double-checked this assumption with the variance ratio test, as in large groups small differences in variance may still result in a significant Levene’s test because of increased power of this test. Effect modification by the individual covariates was tested by assessment of statistical significance of the interaction terms medication*covariate for each of the drug groups of interest. In case of significant interaction (*p* value < 0.1), analyses were stratified. Statistical tests were two-tailed and, apart from the testing of interaction, a *p* value < 0.05 was the criterion for statistical significance.

## Results

### Characteristics

In Table 1 the characteristics of 783 geriatric outpatients are presented. Median number of medications used was 6 [IQR 3–9], prevalence of polypharmacy 65 %, of severe polypharmacy 22 %. Depending on the cut-off value used, prevalence of vitamin D deficiency was 49 % (25(OH)D <50 nmol/l) or 77 % (<75 nmol/l). Of the 152 patients using a vitamin D supplement, a considerable number were still deficient: 17 % at <50 nmol/l and 49 % at <75 nmol/l, respectively and of the 631 non-users, 57 and 83 %, respectively. In the severe polypharmacy subgroup of these non-users, prevalence of deficiency was 73 and 88 %, respectively. Compared to the supplement users, the non-users were younger (≥80 years: 44 % versus 59 %, *p* < 0.01), had a lower risk of malnutrition (MNA screening score 12–14: 62 % versus 45 %, *p* < 0.01), and used fewer medications (median [IQR]: 5 [3–8] versus 9 [6–12], *p* < 0.01).

**Table 1:** Characteristics of a population of 783 Dutch geriatric outpatients, aged 55 years and older

Characteristic	Category	Percentage or median [IQR]	Characteristic	Category	Percentage or median [IQR]
Gender	male	40.1	MMSE (score)		24 [19–27]
	female	59.9		0–18	22.1
Age (year)		79 [73–84]		19–24	31.4
	≥80	46.9		25–30	46.5
BMI (kg/m2)		26.5 [23.6–29.7]	Smoking status	never	64.4
	<21.0	9.4		former	25.0
	21.0–26.9	43.8		current	10.6
	≥27.0	46.8	Serum 25(OH)D (nmol/l)		50 [33–72]
MNA screening^a^ (score)		12 [10–13]		<50	49.4
	0–7	7.9		50–74	27.1
	8-11	33.4		≥75	23.5
	12-14	58.7	Medications^d^ (number)		6 [3–9]
Alcohol use^c^	not/light	85.3		use (≥1)	95.1
	moderate	12.0		polypharmacy (≥5)	64.9
	excessive	2.7		severe polypharmacy (≥10)	21.7
Education level	primary school only	39.3	Vitamin D supplement	use (≥1)	19.4

*ATC* anatomic therapeutic chemical classification, *BMI* body mass index, *IQR* interquartile range, *MMSE* mini mental state examination, *MNA* mini nutrition assessment, *25(OH)D* 25-hydroxyvitamin D

^a^Between 1 August 2011–31 December 2013

^b^In a subpopulation aged ≥65 years, between 21 September 2011–31 December 2013

^c^Alcohol consumption index according to Garretsen [14]

^d^All ATC-coded substances (ATC-coded supplements included)

### Associations between drug use and vitamin D level

Table 2 shows the associations between drug use and serum 25(OH)D adjusted for age and gender, in patients not using a vitamin D supplement (*n* = 631). Associations are expressed as *V*-beta_medication_ (*β*). For the complete results of the further adjustments in the complex model we refer to Table 3 in the electronic supplementary material of the online version of this article.

**Table 2:** Medication^a^ use and associated serum 25(OH)D, adjusted for age and gender^b^, in 631 Dutch geriatric outpatients not using a specific vitamin D supplement

Medication use					Serum 25(OH)D			
ATC code	Medication	Use^c^	No.		Mean^d^ 25(OH)D (nmol/l)	[95 % CI]	Difference^e^ in mean V-25(OH)D	*p* value
Any	Number of medications used		631				−0.0^f^	0.02
Any	Polypharmacy^g^	0	256		48.9	[46.0; 52.0]	−0.3	0.06
		1	375		45.2	[42.9; 47.7]		
Any	Severe polypharmacy^h^							
	males	0	221		50.7	[47.8; 53.7]	−0.7	0.01
		1	47		41.0	[35.5; 46.9]		
	females	0	306		45.3	[42.5; 48.2]	−0.1	0.72
		1	57		44.0	[37.8; 50.8]		
A02BC	Proton pump inhibitors	0	398		47.4	[45.1; 49.9]	−0.1	0.32
		1	233		45.5	[42.5; 48.6]		
A06AD	Osmotically acting laxatives	0	562		47.1	[45.1; 49.1]	−0.3	0.26
		1	69		43.7	[38.4; 49.4]		
A10B	Oral antidiabetics	0	520		47.8	[45.7; 49.9]	−0.4	0.02
		1	111		41.9	[37.8; 46.2]		
A10BA	Biguanides (metformin only)	0	541		47.7	[45.7; 49.8]	−0.5	0.01
		1	90		40.8	[36.3; 45.5]		
A10BB	Sulfonamides and urea derivatives							
	males, age < 80 years^i^	0	148		54.9	[51,3; 58,6]	−1.1	<0.01
		1	20		40.4	[32.4; 49.3]		
	age ≥ 80 years	0	90		42.6	[38.3; 47.2]	−0.2	0.76
		1	10		40.4	[28.5; 54.6]		
	females, age < 80 years	0	167		50.8	[46.8; 55.0]	−0.2	0.69
		1	19		48.2	[37.2; 60.7]		
	age ≥ 80 years	0	163		39.1	[35.6; 42.7]	0.7	0.19
		1	14		48.0	[35.5; 62.4]		
B01AA	Vitamin K antagonists	0	551		47.6	[45.6; 49.6]	−0.5	0.02
		1	80		41.1	[36.3; 46.3]		
B01AC	Platelet aggregation inhibitors	0	404		46.6	[44.3; 49.0]	0.0	0.88
		1	227		46.9	[43.8; 50.1]		
C01AA	Cardiac glycosides (digoxin only)	0	607		47.1	[45.2; 49.0]	−0.7	0.07
		1	24		38.2	[29.9; 47.5]		
C03AA	Thiazide diuretics	0	516		46.8	[44.8; 48.9]	0.0	0.82
		1	115		46.3	[42.0; 50.8]		
C03CA	Loop diuretics							
	age < 80 years	0	324		52.0	[49.3; 54.8]	−0.3	0.45
		1	30		48.5	[40.2; 57.5]		
	age ≥ 80 years	0	217		42.5	[39.6; 45.7]	−0.7	0.01
		1	60		34.4	[29.4; 39.8]		
C03D	Potassium-sparing diuretics							
	males	0	256		49.6	[46.9; 52.3]	−1.1	0.02
		1	12		35.6	[25.8; 47.1]		
	females	0	332		45.1	[42.5; 47.9]	0.0	0.98
		1	31		45.0	[36.5; 54.3]		
C07AB	Selective beta-blocking agents	0	441		47.1	[44.8; 49.4]	−0.1	0.60
		1	190		45.9	[42.6; 49.4]		
C08CA	Dihydropyridines	0	534		46.7	[44.7; 48.7]	0.0	0.92
		1	97		46.9	[42.3; 51.8]		
C09AA	ACE inhibitors	0	474		47.9	[45.7; 50.1]	−0.3	0.04
		1	157		43.3	[39.7; 47.0]		
C09CA	Angiotensin-2 antagonists	0	524		46.1	[44.1; 48.2]	0.3	0.18
		1	107		49.6	[45.0; 54.4]		
C10AA	Statins	0	451		47.4	[45.2; 49.6]	−0.2	0.28
		1	180		45.1	[41.7; 48.6]		
N02BE	Anilides (paracetamol only)	0	538		47.3	[45.3; 49.4]	−0.3	0.13
		1	93		43.3	[38.7; 48.1]		
N05^j^	Benzodiazepines	0	485		46.5	[44.4; 48.7]	0.1	0.73
		1	146		47.3	[43.5; 51.4]		
N06A	Antidepressants							
	males	0	233		48.6	[45.8; 51.4]	0.2	0.49
		1	35		51.4	[44.0; 59.3]		
	females	0	296		45.8	[43.0; 48.8]	−0.3	0.26
		1	67		42.1	[36.4; 48.1]		
N06AA	NSMRIs							
	age < 80 years	0	333		50.8	[48,2; 53,4]	1.1	<0.00
		1	21		68.3	[56.7; 80.9]		
	age ≥ 80 years	0	254		40.8	[38.1; 43.7]	−0.1	0.72
		1	23		39.1	[30.5; 48,8]		
N06AB	SSRIs	0	578		47.4	[45.4; 49.4]	−0.6	0.02
		1	53		39.8	[34.1; 46.0]		

*ATC* anatomic therapeutic chemical Classification, *CI* confidence interval, *PPIs* proton pump inhibitors, *NSMRIs* non-selective monoamine reuptake inhibitors, *SSRIs* selective serotonin reuptake inhibitors, *25(OH)D* 25-hydroxyvitamin D

^a^ATC-coded substances

^b^If interaction with medication: stratification

^c^0 = no use, 1 = use

^d^Squared mean of ‘square-root transformed 25(OH)D’

^e^Difference in mean ‘square root transformed 25(OH)D’ between users and non-users of a medication (which is equivalent to the regression coefficient β of an association between drug use and 'square root transformed 25(OH)D')

^f^-0.043 = regression coefficient β of the association between the number of medications used and ‘square root transformed 25(OH)D’ (which is equivalent to the difference in mean ‘square root transformed 25(OH)D’ between users and non-users of a medication)

^g^Use of ≥5 medications concomitantly

^h^Use of ≥10 medications concomitantly

^i^Independent *t* test because of unequal variances

^j^N05BA, N05CD or N05CF

The majority of statistical significant associations were inverse associations. In the simple model, adjusting for age and gender, we found negative associations for the number of medications used, polypharmacy, severe polypharmacy, use of oral antidiabetics, metformin, SUDs, vitamin K antagonists, cardiac glycosides, loop diuretics in subjects age ≥ 80 years, potassium-sparing diuretics, ACE inhibitors, and the antidepressive SSRIs. By contrast, the association with the antidepressive NSMRIs was positive in subjects < 80 years. The most extreme differences between adjusted mean 25(OH)D level of users and non-users of a medication were −14.5 nmol/l for SUDs and +17.5 nmol/l for NSMRIs.

After further adjustment for BMI, MMSE score, and use of multivitamins, statistical significance of associations with oral antidiabetics, metformin, and SSRIs disappeared. The inverse association with vitamin K antagonists was only borderline significant in subjects with a BMI ≥27.0 (*β* = −0.5, *p* = 0.07). An inverse association with thiazide diuretics became statistically significant for subjects with a BMI <21.0 (*β* = −1.5, *p* = 0.01), just as a positive association for angiotensin-2 antagonists in patients with MMSE scores between 0-24 (*β* = 0.5, *p* = 0.02). The most extreme differences in adjusted mean 25(OH)D level were −19.3 nmol/l for thiazide diuretics and +16.8 nmol/l for NSMRIs.

## Discussion

Our study population consisted of 783 community-living older people who visited the geriatric outpatient clinic for various complaints. In the total population, polypharmacy and vitamin D deficiency were highly prevalent. Amongst users of vitamin D supplements, vitamin D deficiency was still prevalent. In patients not using supplemental vitamin D, inverse associations with serum 25(OH) were observed for the number of medications used, (severe) polypharmacy, antidiabetics, cardiac glycosides, diuretics, ACE inhibitors, and SSRIs; positive associations for angiotensin-2 antagonists and NSMRIs.

In the following discussion of possible mechanisms behind observed associations, confounding by indication should be kept in mind: the entanglement of medication and indication for its prescription as precipitating factors.

To start with the inverse associations between the *number of medications* used and *polypharmacy* and vitamin D level, these were also observed in the only other cross-sectional study investigating these relationships [18]. Such inverse associations were not unexpected, as polypharmacy is associated with frailty [21], which in turn is a predictor of low circulating 25(OH)D [22].

A lack of relationship with use of *PPIs* was confirmed by a quasi-experimental study (*n* = 21) [23] and a prospective cohort study (n = 58) [24]. A cross-sectional study (*n* = 737) detected a borderline significant inverse association for which no explanation was given [18].

The absence of a significant association for *osmotically acting laxatives* could not be compared with literature data as no other publications were identified.

In three cross-sectional studies a negative association with *oral antidiabetics* was also observed (*n* = 737, *n* = 407, *N* = 11256) [18, 25, 26]. At the same time, a previous quasi-experimental study (*n* = 19) and a cross-sectional study (*n* = 698), did not show these associations [27, 28]. Obviously, not only medication itself might be an explanation, but also the indication for its prescription. This confounding by disease is supported by a meta-analysis of prospective studies investigating 25(OH)D and type 2 diabetes [29]. Underlying mechanisms might include a decreased insulin sensitivity related with increased parathyroid hormone levels which, in turn, are associated with vitamin D deficiency [30]. Another explanation might be vitamin D deficiency being mediated by inflammatory mediators which are associated with the presence of excess visceral fat, a risk factor for diabetes mellitus type-2 [10].

Lower 25(OH)D levels in users of *vitamin K antagonists* were also seen in a Dutch cross-sectional study in 514 females;[18] and in a German cross-sectional study in 7553 males [26]. In three other cross-sectional studies (*n* = 116, *n* = 48, *n* = 127) and one prospective cohort study (*n* = 167), no associations were observed [31–34].

Associations with *platelet aggregation inhibitors* were contradictory in literature. Similar to our study, one cross-sectional study (*n* = 1301, *n* = 737) did not show an inverse relationship, [18] while three others did (*n* = 11256, *n* = 2016, *n* = 459) [26, 35, 36]. Confounding by indication might lie behind the suggestion that elevated inflammatory markers link hypovitaminosis D with increased risk of cardiovascular disease [36].

Opposite to our results, an inverse relationship with cardiac glycosides was not observed in two cross-sectional studies investigating digoxin (*n* = 1301, *n* = 11256) [18, 26].

Also in other studies observed associations with *thiazide diuretics* were mixed. Two cross-sectional studies (*n* = 1301, *n* = 737; *n* = 66) did not report any significant association [18, 37]. Contrarily, a small randomised placebo-controlled cross-over trial (*n* = 23) reported a dose-dependent increase in 25(OH)D levels, [38] while a third cross-sectional study (*n* = 302) reported an inverse association [39]. This might be confounded by indication as hypertension is inversely associated with vitamin D status [40].

Lower 25(OH)D levels amongst users of *loop diuretics* were also determined in two cross-sectional studies (*n* = 1301, *n* = 280), [18, 41] In the first study, results were significant when adjusted for age and gender; when number of chronic diseases was added, significance disappeared. However, in a complex model with six extra covariates the association was again significant. In two other cross-sectional studies (*n* = 302, *n* = 77) associations were not statistically significant [39, 42].

Our observation of an inverse association with *potassium-sparing diuretics* was not supported by one other cross-sectional study (*n* = 1301) [18]

No association with *selective beta-blocking agents* was found in two cohorts of a cross-sectional study (*n* = 1301, *n* = 737) [18].

Similar to our results, no difference in 25(OH)D levels was reported in a small cross-sectional study (*n* = 22) investigating *dihydropyridines* [43]. This was also the case in two other, larger cross-sectional studies (*n* = 1301, *n* = 11256) investigating the broader therapeutic subgroup of calcium blocking agents [18, 26]. However, in a second cohort of one of these studies (*n* = 737) a statistically significant inverse association was identified [18]. It is hypothesised that activation of the nuclear pregnane X receptor by calcium blockers might lead to catabolism of 25(OH)D [44].

Lower 25(OH)D levels in users of *ACE inhibitors* were also observed in a German and a Dutch cross-sectional study (*n* = 11256, *n* = 737) [ref 15, 22], while in a second cohort of the Dutch study (*n* = 1301) [18] and in two quasi-experimental studies (*n* = 73, *n* = 60) [45, 46] no relationship was shown. A third quasi-experimental study reported higher levels in users of quinapril (*n* = 23) and no change in users of enalapril (*n* = 23) [47]. It cannot be excluded that an inverse association reflects the inverse relationship between low 25(OH)D level and the indication for prescription: high blood pressure^29^ and associated comorbidities of diabetes [29].

Our finding of a positive association with *angiotensin-2 antagonists* was not detected in two other cross-sectional studies (*n* = 11256, *n* = 31) [26, 48].

Similar to our study, no association with *statins* was detected in two regression models of a cross-sectional study after multiple adjustments (*n* = 737); in a third model of this study, a borderline significantly inverse association was found after adjustment for age and gender only [18]. In a large cross-sectional and small cohort study (*n* = 11256, *n* = 208) associations observed were positive [26, 49]. Studies investigating individual statins reported null or positive associations [9]. Several mechanisms are suggested for a positive association: an increase in level of precursors for vitamin D synthesis in the skin by inhibition of the HMG-CoA reductase, [50] competition of statins with the metabolising enzyme CYP3A4, [51], or inhibition by statins of vitamin D-consuming inflammatory processes [27].

The absence of a significant association with *benzodiazepines* was also reported in two other cross-sectional studies (*n* = 737, *n* = 589) [18, 52]. In a second cohort of the first study, a significant inverse association was observed (*n* = 1301) [18].

The finding of an inverse relationship with *antidepressants* in one other cross-sectional study (*n* = 589) [52] supports our finding of lower 25(OH)D levels in users of *SSRIs*. An inverse association might be explained by inhibition of 25(OH)D synthesis through inhibition of the CYP3A4 enzyme [53]. The inverse relationship between depression and 25(OH)D reported in literature [54] does not match with our results for users of *NSMRIs*, who had higher 25(OH)D levels compared to non-users in the complex model.

The finding that a considerable part of the reported users of vitamin D supplements were still deficient is noteworthy. Possible explanations could lie in the type and quality of vitamin D supplements, dosing guidelines, duration of use, compliance, and interindividual differences in pharmacodynamics and pharmacokinetics in our group of patients, but these remain to be investigated further.

Major limitation of our study is the cross-sectional design, which implies that no conclusions can be drawn about causal relationships. A critical remark has also to be made about our interpretation of cross-sectional. Laboratory measurements of 25(OH)D were included until four months preceding the first visit of the outpatient clinic; 95 % of these assays were measured on the day of first visit. Underlying considerations were the following. Firstly, the pragmatic reason that physicians did not request vitamin D testing within four months following a previous measurement in the hospital. Secondly, the assumption that the investigated drugs were already being used for a longer period, so also at the moment of a previous vitamin D assessment. One more critical remark must be made, on the HPLC assay used for 25(OH)D measurement. This is a less accurate method compared to the golden standard liquid chromatography-mass spectrometry. Another limitation of the study is the registration of the drugs used, which was dependent on the cooperation, memory, and assistance of the patients or their accompanying persons, and the accurateness of drug registration by pharmacies. Furthermore, although patients were also routinely asked for drug therapy compliance, no drug analysis was undertaken to confirm their response or that of their helper. This, combined with the fact that registration in the patient files was not standardised, was reason not to include compliance as a potential covariate. Uncertainty about drug compliance also influences the effect of use of a vitamin D supplement. Long-term formulations could be an option to improve this. Also important to mention is that we did not adjust for confounding by indication. Reasons were the complexity of this confounder and lacking data. Finally, we note that one or more of the statistically significant results may be regarded as chance findings. The large number of medication groups increases the possibility of false-positive results.

Despite these reservations, our study has several noteworthy strengths. It adds to the data of the relatively unexplored field of drug-nutrient interactions and reflects the typical population of community-dwelling older people visiting a non-academic regional hospital. That we investigated a large number of drug groups which are frequently used by older people further contributes to the quality of the study. Finally, the ATC-coding is a major strength; it enabled us to investigate drug and supplement use in a systematic way. Moreover, as far as we know, in studies investigating drug use and circulating 25(OH)D, we are the first to discriminate between users and non-users of supplemental vitamin D.

## Conclusion

Drug use should be recognised as a determinant of vitamin D status. The still high prevalence of vitamin D deficiency in subjects using a vitamin D supplement requires further investigation.

## Electronic supplementary material

Below is the link to the electronic supplementary material.

ESM 1 In the electronic supplementary material section of the European Journal of Clinical Pharmacology’s website, more details are given on assessment of vitamin D status. In Table 3 results are presented of multiple linear regression analysis of the association between drug use and vitamin D level adjusted for age, gender, BMI, MMSE score, and use of a multivitamin supplement in geriatric outpatients not using a specific vitamin D supplement. (DOCX 51.3 kb)
