# Supplementary material for: Vitamin D deficiency as adverse drug reaction? A cross-sectional study in Dutch geriatric outpatients
Source: Eur J Clin Pharmacol. 2016 Feb 12;72:605–14. doi: 10.1007/s00228-016-2016-2 (PMC4834096; doi:10.1007/s00228-016-2016-2)
Supplement: Supplementary file 1 — In the electronic supplementary material section of the European Journal of Clinical Pharmacology’s website, more details are given on assessment of vitamin D status. In Table 3 results are presented of multiple linear regression analysis of the association between drug use and vitamin D level adjusted for age, gender, BMI, MMSE score, and use of a multivitamin supplement in geriatric outpatients not using a specific vitamin D supplement. (DOCX 51.3 kb) [file 228_2016_2016_MOESM1_ESM.docx]

# Electronic supplementary material

**Article title**

Vitamin D deficiency as adverse drug reaction? A cross-sectional study in Dutch geriatric outpatients

**Journal name**

European Journal of Clinical Pharmacology

**Corresponding author**

Anne Claire B. van Orten-Luiten, Division of Human Nutrition, Wageningen University, Wageningen, The Netherlands; email: [wout.vanorten-luiten@wur.nl](mailto:wout.vanorten-luiten@wur.nl)

**Co-authors**

André Janse, Rosalie A.M. Dhonukshe-Rutten, Renger F. Witkamp

**Content**

Details on laboratory measurement of 25(OH)D concentrations by the laboratory of Hospital Gelderse Vallei in Ede are given plus the results of multiple linear regression analysis of the association between drug use and vitamin D level in geriatric outpatients not using a specific vitamin D supplement (Table 3). Confounders were included in the regression model if they changed the unstandardised regression coefficient ≥10% after being added (stepwise method).

**Assessment of vitamin D status**

Vitamin D status was measured in the laboratory of the Gelderse Vallei Hospital in Ede, The Netherlands, using the 25-OH-Vitamin D3/D2 Reagent Kit for HPLC analysis according to the manufacturer’s instructions (Chromsystems Instruments & Chemicals, Gräfelfing, Germany). Chromsystems uses NIST 972a Standard reference material when setting values for its controls.

- Once a week serum 25(OH)D3 is quantified
- After clotting and centrifugation of serum separator tubes, serum samples are stored deep frozen at -20 degrees Celsius
- Stability of processed samples is 3 days at room temperature or 7 days when stored between 2-8 degrees Celsius
- Recovery of 25(OH)D3 is 86%
- Measuring range: vitamin 25(OH)D3 levels can be accurately measured between 6.2 and 1248 nmol/L. Levels outside this range are reported as <6.2 nmol/L and >1248 nmol/L.
- Accuracy of measurements is monitored by using the Chromsystems controls which are traceable to the NIST reference material at 2 different levels. Results are as follows: at level 33.9 nmol/L we measured 33.0 nmol/L (97.3%)*; at level 135.0 nmol/L we measured 127.0 nmol/L (94.1%). (*recovery)
- Precision of measurements is 5,5% at level 33 nmol/l, 4,3% at level 89.5 nmol/l, and 6,8% at level 127 nmol/l.

| **Table 3 M**edication^a^ use and associated serum 25(OH)D, adjusted for age, gender, BMI, MMSE, and use of a multivitamin supplement^b^, in 602 Dutch geriatric outpatients^c^ not using a specific vitamin D supplement | | | | | | | | | |
| --- | --- | --- | --- | --- | --- | --- | --- | --- | --- |
| Medication use | | | |  | Serum 25(OH)D | | | | |
| ATC  code | Medication^a^ | Use^d^ | No. |  | Mean^e^  25(OH)D  (nmol/l) | [95% CI] | | Difference^e^ in mean  Ѵ-25(OH)D | P-value |
|  |  |  |  |  |  |  | |  |  |
| Any | Number of medications used |  | 602 |  |  |  |  | -0.0^g^ | 0.01 |
|  |  |  |  |  |  |  |  |  |  |
| Any | Polypharmacy^h^ | 0 | 247 |  | 48.8 | [45.9; | 51.9] | -0.3 | 0.06 |
|  |  | 1 | 355 |  | 45.1 | [42.8; | 47.5] |  |  |
| Any | Severe polypharmacy^i^ |  |  |  |  |  |  |  |  |
|  | males | 0 | 209 |  | 51.0 | [48.2; | 53.9] | -0.7 | <0.01 |
|  |  | 1 | 46 |  | 41.3 | [35.8; | 47.1] |  |  |
|  | females | 0 | 293 |  | 45.0 | [42.3; | 47.8] | -0.1 | 0.71 |
|  |  | 1 | 54 |  | 43.7 | [37.4; | 50.4] |  |  |
| A02BC | Proton pump inhibitors | 0 | 381 |  | 47.5 | [45.2; | 49.8] | -0.2 | 0.23 |
|  |  | 1 | 221 |  | 45.2 | [42.2; | 48.2] |  |  |
| A06AD | Osmotically acting laxatives | 0 | 537 |  | 46.9 | [44.9; | 48.8] | -0.2 | 0.49 |
|  |  | 1 | 65 |  | 44.8 | [39.5; | 50.4] |  |  |
| A10B | Oral antidiabetics | 0 | 495 |  | 47.1 | [45.1; | 49.2] | -0.2 | 0.28 |
|  |  | 1 | 107 |  | 44.4 | [40.2; | 48.9] |  |  |
| A10BA | Biguanides (metformin only) | 0 | 515 |  | 47.2 | [45.2; | 49.2] | -0.3 | 0.30 |
|  |  | 1 | 87 |  | 43.2 | [38.7; | 48.1] |  |  |
| A10BB | Sulfonamides & urea derivatives |  |  |  |  |  |  |  |  |
|  | males. age <80 yr | 0 | 141 |  | 54.3 | [50.8; | 57.9] | -0.7 | 0.06 |
|  |  | 1 | 19 |  | 44.3 | [35.7; | 53.9] |  |  |
|  | age ≥80 yr | 0 | 86 |  | 43.1 | [38.8; | 47.7] | -0.0 | 0.94 |
|  |  | 1 | 9 |  | 42.5 | [29.8; | 57.5] |  |  |
|  | females. age <80 yr | 0 | 159 |  | 49.4 | [45.5; | 53.5] | 0.3 | 0.51 |
|  |  | 1 | 19 |  | 53.7 | [42.1; | 66.8] |  |  |
|  | age ≥80 yr | 0 | 156 |  | 39.0 | [35.7; | 42.5] | 0.7 | 0.15 |
|  |  | 1 | 13 |  | 48.6 | [36.3; | 62.8] |  |  |
| B01AA | Vitamin K antagonists |  |  |  |  |  |  |  |  |
|  | BMI <21.0 | 0 | 49 |  | 49.8 | [42.2; | 58.0] | -1.2 | 0.12 |
|  |  | 1 | 8 |  | 34.2 | [19.8; | 52.5] |  |  |
|  | BMI 21.0-26.9 | 0 | 226 |  | 47.7 | [44.7; | 50.8] | -0.4 | 0.23 |
|  |  | 1 | 36 |  | 42.7 | [35.6; | 50.4] |  |  |
|  | BMI ≥27.0 | 0 | 248 |  | 47.0 | [44.3; | 49.8] | -0.5 | 0,07 |
|  |  | 1 | 35 |  | 39.9 | [33.3; | 47.0] |  |  |
| B01AC | Platelet aggregation inhibitors | 0 | 385 |  | 46.6 | [44.3; | 48.9] | 0.0 | 0.96 |
|  |  | 1 | 217 |  | 46.7 | [43.7; | 49.8] |  |  |
| C01AA | Cardiac glycosides (digoxin only) | 0 | 578 |  | 47.0 | [45.2; | 48.9] | -0.7 | 0.04 |
|  |  | 1 | 24 |  | 37.5 | [29.7; | 46.3] |  |  |
| C03AA | Thiazide diuretics |  |  |  |  |  |  |  |  |
|  | BMI <21.0 | 0 | 50 |  | 50.1 | [42.6; | 58.1] | -1.5 | 0.06 |
|  |  | 1 | 7 |  | 30.8 | [16.6; | 49.3] |  |  |
|  | BMI 21.0-26.9 | 0 | 223 |  | 46.0 | [43.0; | 49.1] | 0.5 | 0.09 |
|  |  | 1 | 39 |  | 53.1 | [45.6; | 61.2] |  |  |
|  | BMI ≥27.0 | 0 | 220 |  | 46.7 | [43.8; | 49.7] | -0.2 | 0.40 |
|  |  | 1 | 63 |  | 44.1 | [38.9; | 49.6] |  |  |
| C03CA | Loop diuretics |  |  |  |  |  |  |  |  |
|  | age <80 yr | 0 | 308 |  | 51.6 | [48.9; | 54.2] | -0.2 | 0.61 |
|  |  | 1 | 30 |  | 49.2 | [41.1; | 58.0] |  |  |
|  | age ≥80 yr | 0 | 205 |  | 42.5 | [39.5; | 45.5] | -0.5 | 0.03 |
|  |  | 1 | 59 |  | 35.7 | [30.7; | 41.1] |  |  |
| C03D | Potassium sparing diuretics |  |  |  |  |  |  |  |  |
|  | males | 0 | 243 |  | 49.8 | [47.2; | 52.5] | -0.9 | 0.04 |
|  |  | 1 | 12 |  | 37.5 | [27.7; | 48.8] |  |  |
|  | females | 0 | 316 |  | 44.5 | [41.9; | 47.2] | 0.2 | 0.51 |
|  |  | 1 | 31 |  | 47.6 | [39.1; | 56.8] |  |  |
| C07AB | Selective beta blocking agents | 0 | 418 |  | 46.5 | [44.4; | 48.7] | 0.0 | 0.88 |
|  |  | 1 | 184 |  | 46.8 | [43.6; | 50.2] |  |  |
| C08CA | Dihydropyridines | 0 | 507 |  | 46.4 | [44.5; | 48.4] | 0.1 | 0.64 |
|  |  | 1 | 95 |  | 47.6 | [43.1; | 52.4] |  |  |
| C09AA | ACE inhibitors | 0 | 448 |  | 47.7 | [45.6; | 49.9] | -0.3 | 0.05 |
|  |  | 1 | 154 |  | 43.5 | [40.0; | 47.2] |  |  |
| C09CA | Angiotensin-2 antagonists |  |  |  |  |  |  |  |  |
|  | MMSE 0-24 | 0 | 281 |  | 42.5 | [40.0; | 45.2] | 0.5 | 0.04 |
|  |  | 1 | 62 |  | 49.2 | [43.4; | 55.4] |  |  |
|  | MMSE 25-30 | 0 | 236 |  | 50.8 | [47.6; | 54.0] | 0.0 | 0.94 |
|  |  | 1 | 45 |  | 51.1 | [43.9; | 58.9] |  |  |
| C10AA | Statins | 0 | 431 |  | 47.2 | [45.1; | 49.4] | -0.2 | 0.31 |
|  |  | 1 | 171 |  | 45.1 | [41.7; | 48.6] |  |  |
| N02BE | Anilides (paracetamol only) | 0 | 515 |  | 47.2 | [45.2; | 49.2] | -0.3 | 0.17 |
|  |  | 1 | 87 |  | 43.6 | [39.0; | 48.4] |  |  |
| N05^j^ | Benzodiazepines | 0 | 466 |  | 46.4 | [44.4; | 48.5] | 0.1 | 0.70 |
|  |  | 1 | 136 |  | 47.3 | [43.5; | 51.3] |  |  |
| N06A | Antidepressants |  |  |  |  |  |  |  |  |
|  | males | 0 | 222 |  | 48.6 | [45.9; | 51.4] | 0.3 | 0.24 |
|  |  | 1 | 33 |  | 53.4 | [46.0; | 61.2] |  |  |
|  | females | 0 | 283 |  | 45.3 | [42.5; | 48.1] | -0.2 | 0.44 |
|  |  | 1 | 64 |  | 42.7 | [37.1; | 48.7] |  |  |
| N06AA | NSMRIs |  |  |  |  |  |  |  |  |
|  | age <80 yr | 0 | 319 |  | 50.5 | [47.9; | 53.0] | 1.1 | 0.01 |
|  |  | 1 | 19 |  | 67.3 | [55.7; | 80.0] |  |  |
|  | age ≥80 yr | 0 | 242 |  | 41.0 | [38.4; | 43.8] | -0.1 | 0.73 |
|  |  | 1 | 22 |  | 39.4 | [31.0; | 48.8] |  |  |
| N06AB | SSRIs | 0 | 551 |  | 47.0 | [45.1; | 48.9] | -0.3 | 0.19 |
|  |  | 1 | 51 |  | 42.7 | [36.9; | 48.9] |  |  |
|  | | | | | | | | | |
| *ATC* anatomic therapeutic chemical Classification; *BMI* body mass index. CI confidence interval; *MMSE* mini mental state examination. *NSMRIs* non-selective monoamine reuptake inhibitors; *SSRIs* selective serotonin reuptake inhibitors. *PPIs* proton pump inhibitors; *25(OH)D* 25-hydroxyvitamin D  ^a^ ATC-coded substances  ^b^ If interaction with medication: stratification  ^c^ 29 subjects were not included in the complex model because of missing data  ^d^ 0 = no use, 1 = use  ^e^ Squared mean of ‘square root transformed 25(OH)D’  ^f^ Difference in mean ‘square root transformed 25(OH)D’ between users and non-users of a medication (which is equivalent to the regression coefficient β of an association between drug use and ‘square root transformed 25(OH)D’)  ^g^ -0.049 = regression coefficient β of the association between the number of medications used and ‘square root transformed 25(OH)D’ (which is equivalent to the difference in mean ‘square root transformed 25(OH)D’ between users and non-users of a medication)  ^h^ Use of ≥5 medications concomitantly.  ^i^ use of ≥10 medications concomitantly  ^j^ N05BA, N05CD or N05CF | | | | | | | | | |
